# Perceived Intensification in Harmful Algal Blooms Is a Wave of Cumulative Threat to the Aquatic Ecosystems

**DOI:** 10.3390/biology11060852

**Published:** 2022-06-02

**Authors:** Syed Shabi Ul Hassan Kazmi, Neelamanie Yapa, Samantha C. Karunarathna, Nakarin Suwannarach

**Affiliations:** 1 Research Center of Microbial Diversity and Sustainable Utilization, Chiang Mai University, Chiang Mai 50200, Thailand; shabiulhassansyed5@gmail.com; 2 College of Marine Life Sciences, Ocean University of China, Qingdao 266003, China; 3 Department of Biological Sciences, Faculty of Applied Sciences, Rajarata University of Sri Lanka, Mihintale 50300, Sri Lanka; pnyapa40@yahoo.co.uk; 4 Center for Yunnan Plateau Biological Resources Protection and Utilization, College of Biological Resource and Food Engineering, Qujing Normal University, Qujing 655011, China

**Keywords:** aquatic pollution, ecotoxicity, ecosystems, eutrophication, harmful algal blooms

## Abstract

**Simple Summary:**

Harmful algal blooms (HABs) are a serious threat to aquatic environments. The intensive expansion of HABs across the world is a warning signal of environmental deterioration. Global climatic change enforced variations in environmental factors causing stressed environments in aquatic ecosystems that favor the occurrence, distribution, and persistence of HABs. Perceived intensification in HABs increases toxin production, affecting the ecological quality as well as serious consequences on organisms including humans. This review outlines the causes and impacts of harmful algal blooms, including algal toxicity, grazing defense, management, control measures, emerging technologies, and their limitations for controlling HABs in aquatic ecosystems.

**Abstract:**

Aquatic pollution is considered a major threat to sustainable development across the world, and deterioration of aquatic ecosystems is caused usually by harmful algal blooms (HABs). In recent times, HABs have gained attention from scientists to better understand these phenomena given that these blooms are increasing in intensity and distribution with considerable impacts on aquatic ecosystems. Many exogenous factors such as variations in climatic patterns, eutrophication, wind blowing, dust storms, and upwelling of water currents form these blooms. Globally, the HAB formation is increasing the toxicity in the natural water sources, ultimately leading the deleterious and hazardous effects on the aquatic fauna and flora. This review summarizes the types of HABs with their potential effects, toxicity, grazing defense, human health impacts, management, and control of these harmful entities. This review offers a systematic approach towards the understanding of HABs, eliciting to rethink the increasing threat caused by HABs in aquatic ecosystems across the world. Therefore, to mitigate this increasing threat to aquatic environments, advanced scientific research in ecology and environmental sciences should be prioritized.

## 1. Introduction

Over the last few decades, there has been an escalating and worrisome trend in the incidence of phenomena known as “Harmful Algal Blooms” (HABs). This term is very broad, covering the diverse nature of blooms, but, most specific, HAB is the word related mostly to the entities that cause harm, either by toxin production or cell accumulation in larger masses altering the normal food web dynamics [1,2]. Freshwater, brackish water, and marine ecosystems are usually inhabited by a diverse set of algal species including diatoms, flagellates, dinoflagellates, cyanobacteria, and chrysophyte that can cause the harmful blooms and may produce toxins that can harm other inhabiting organisms and human beings. With exposure to certain environmental conditions, such as rising temperature and nutrient accumulation (e.g., nitrates and phosphates), various phytoplankton species can grow excessively and create larger masses of algal blooms [3,4,5,6]. The events of HABs are characterized by the explosion and sporadic dominance of toxic or harmful algal species. In some cases, these cell masses gained higher abundances and their pigments discolor the freshwater, commonly called a “red tide” or “brown tide” [7,8]. However, among these blooms, many species do not have dominant cell numbers and cannot cause water discoloration but are still harmful because of potent toxin production leading to devastating effects [1].

Harmful algal blooms (HABs) especially cover the algal species that grow extensively on a global scale and can cause substantial damage while producing toxic or harmful effects on humans, fish, shellfish, marine animals, and birds [9,10]. Most of the harmful bloom-forming species belong to six different cyanobacterial and algal groups including cyanophytes, diatoms, dinoflagellates, haptophytes, raphidophytes, and pelagophytes, that widely differ in their morphological, physiological, and ecological characteristics [11]. Cyanobacterial bloom-forming species include *Dolicho spermum*, *Aphanizomenon*, *Cylindrospermopsis*, *Gloeotrichia*, *Microcystis*, *Nodularia*, *Planktothrix*, *Pseudanabaena*, *Synechococcus*, *Trichodesmium*, *Woronichinia*, and benthic HABs *Lyngbya*, *Oscillatoria*, *Phormidium*, and *Scytonema* [12]. Some of these genera thrive in both fresh and brackish water while some are widespread in marine ecosystems. Recurring cyanobacterial harmful algal blooms (cHABs) are evident in some of the world’s largest inland freshwater ecosystems, Lake Victoria (Africa), Lake Erie and Lake Michigan (USA–Canada), Lake Okeechobee (Florida, USA), Lake Pontchartrain (Louisiana, USA), and Lake Taihu (China) [13,14]. Whereas, species succession from small to large diatoms and dinoflagellates usually dominates the aquatic bodies over the spring to summer seasons [15,16]. However, some of the dinoflagellates *Dinophysis acuminata*, *Prorocentrum minimum*, and the diatom *Pseudo-nitzschia multiseries* expand aquatic ecosystems in late spring or summer seasons [13,17].

Increased amounts of nutrients (e.g., eutrophication) together with several other factors, such as suitable temperature, light intensity, and movement of species can contribute to HAB formation [18,19]. Eutrophication and other physical, biological, and chemical factors are responsible for modulating the effects of increased nutrient loading in aquatic environments, influencing HAB population dynamics [6]. The climatic changes including regional and global warming may also favor the initiation, magnitude, duration, and distribution of HABs by disturbing dynamics of programmed algal cell death [20]. The spatial variations in climatic patterns are expected to expand the geographic ranges of benthic harmful algal blooms (BHABs) [21,22,23]. Whereas, the strong alterations of atmospheric and oceanic circulation dynamics in the southeastern Pacific Ocean were also linked to massive HABs in 2016 [24]. It is assumed that the surface temperatures in the world’s oceans are projected to warm by 0.4–1.4 °C by the mid-21st century, causing many tropical and sub-tropical harmful dinoflagellate genera such as *Gambierdiscus*, *Fukuyoa*, and *Ostreopsis* BHABs to exhibit higher growth rates over much of their current geographic range, resulting in higher population densities [25]. Moreover, the coastal waters have experienced progressive warming, acidification, and deoxygenation that will intensify this century, at the same time the impacts on the ecosystem from HABs have all increased over the past few decades [26]. Therefore, the wave of increasing threat by HABs in aquatic environments is accelerating and it needs to be explored at a larger scale for aquatic environment sustainability.

Expansion of global HABs has gained enough recognition; however, the bloom formation events were not sufficiently studied even though many countries were facing the bewildering array of their impacts [27,28]. Various bloom-forming dinoflagellate species have been documented as common species such as *Prorocentrum minimum* expanding throughout the world coastlines with escalating eutrophication [29]. Likewise, in the US HABs significantly increased their frequency and abundance leading to high aquatic contamination and bewildering effects in various parts from the 1980s to 2018 [30,31,32,33]. Similarly, in Europe and Asia, algal bloom formation is increasing with more potent toxic effects than in previous decades [34]. Whereas, in the past decade Arabian regions also faced the massive expansion of HABs [35]. However, these challenges are mirrored globally with recent examples, including an expansion of various harmful algal species such as *Pseudochattonella* cf. *verruculosa* in the south and *Alexandrium catenella* in the north in Chile, leading to massive fishery cessations in 2016, and disruption by *Alexandrium catenella* in Tasmania, southeastern Australia, from 2012 to 2017 of the fishing industry in poorly monitored coastal waters [24,33,36]. HABs significantly affect the water quality, by damaging its aesthetic value, causing oxygen deficiency, aquatic toxification, and affecting the biodiversity by higher mortalities of marine biota [37,38,39,40,41]. Apart from the toxic effects, HABs have also stressed socio-economic infrastructure including food trade, recreation, tourism, and sports [42]. The high degree of spatio-temporal heterogeneity in species composition, non-point source factors responsible for HABs formation, and imperfect biomass–toxicity relationships have caused a major challenge in understanding, forecasting, mitigating, and controlling the events of HABs across the aquatic environments. The most critical component in predicting, understanding, and addressing the substantial socio-economic, ecological, and human health concerns posed by HABs lies in research studies and management or monitoring communities capable of: (a) understanding the general and specific factors and mechanisms contributing to the formation of HABs, (b) vigorously detecting the biomass changes, especially most dominant and hazardous taxa, and (c) indicating the early toxicity information and possible remedies of mitigation to minimize the economic losses. To highlight the threat of HABs to aquatic environments, however, this review summarizes some major factors influencing the formation of HABs, toxicity, economic and ecological impacts, human health impacts, grazing defense, and management of HABs.

## 2. Factors Influencing the Formation of Harmful Algal Blooms (HABs)

There is a growing body of scientific literature focused on the influence of factors such as climatic change and eutrophication individually. However, due to the lack of literature, there is a concern for climatic change and its associated factors to be summarized along with other factors in one study. Therefore, in this review, an extensive effort was made to summarize the literature about various exogenous factors influencing the formation of HABs.

### 2.1. Interaction between Climatic Changes and Other Stressors

Griffith and Gobler [43] discussed climate change as a co-stressor to HABs, causing warming, acidification, and deoxygenation to provoke intensifying impacts of HABs in aquatic ecosystems. For deep understanding, the authors demonstrated the co-occurrence of climate change in coastal zones and co-effects of climate change stressors and HABs on aquatic life. Their review discussed the ecological and physiological framework for considering HABs as a climate change co-stressor and considers the consequences of their combined occurrence for coastal ecosystems. It also emphasized the importance of the inclusion of HAB species in experiments and monitoring programs that take into account the effects of multiple climate change stressors, as this will provide a more ecologically relevant perspective of the ecosystem structure and function of climate-altered systems in the future.

Wells et al. [44] demonstrated linkages between fisheries and aquaculture with experimental work specifically highlighting the application of experimental and field studies, extended observational programs, and retrospective studies, for an in-depth understanding of the socio-economic effects of HABs and climate change. Further, temperature variations, nutrient availability, precipitation, ocean acidification, and the physical structure of the water column, all influence the composition, productivity, and global range of phytoplankton accumulations. However, large uncertainty remains about how these climate drivers’ integration strengthens the shape of HABs in the future. They also mentioned that cyanobacterial HABs, benthic HABs, and HAB effects on fisheries are all concerned with the effects of temperature, light, stratification, salinity, storm severity, ocean acidification, nutrients, and grazer drivers on microalgae. Gobler [45] demonstrated that HABs displayed range expansion since 1980 and increased frequency in response to climatic change. These changes have brought increasing trends in HABs, which are partly due to the effects of thermal stress in marine ecosystems, particularly marine heatwaves, oxygen loss, ocean warming, increasing pollution intensity, and eutrophication. Consequently, these diversified HAB trends have negative impacts on human health, the local economy, tourism, and food security issues as well. Furthermore, the report outlined a number of connections between thermal waves and HABs. However, it has also been documented that anthropogenic interventions, climatic change, and individual events driven by regional, local, and global drivers promoted the trends of HABs globally. Ralston and Moore [46] discussed the utility of statistical models as well as the strengths and weaknesses of these models to model the HAB responses to climatic changes. They evaluate the HAB models with extensive observations and formulate suggestions for researchers to move forward in developing models that are more robust to project the impacts of climate change on HABs. Suggestions to assess the prolonged trends of HABs associated with climatic changes were also drawn from this work. Their review outlined that the statistical models widely employed for forecasting the near-term HABs and resource management, according to their analysis, are not well adapted for longer-term projections since forcing circumstances may differ from previous observations. Process-based models, on the other hand, are difficult to parameterize, require extensive calibration, and are more complicated, but they may predict the HAB response mechanistically under varying conditions. However, process-based models are vulnerable to failure if crucial processes emerge because of climate change that was not recognized during the development of the model. Furthermore, as resource managers and policymakers demand more forecasts of HAB consequences for both short and long time periods, modeling research based on HAB response to climate change will certainly expand. Such HAB models will be critical in informing the development of solution strategies to minimize the public health and socioeconomic impacts as well as build socio-ecological system resilience to future HABs.

Hennon and Dyhrman [47] discussed that during the last 20 years omics techniques such as transcriptomics, metabolomics, omics, genomics, and proteomics transformed the landscape data of several fields including HABs studies. Technological advancements have provided a breakthrough to create many omics datasets that are complementary and publicly available and provide new insights on HAB formation and toxin production. Genomic analysis on the other hand has been utilized to uncover the differences in nutritional requirements and toxicity mechanisms. Whereas, proteomics and transcriptomics have been employed to explore the responses of HAB species to environmental stressors while metabolomics can reveal toxicity and allelopathy mechanisms. The omics data, however, could be leveraged for improvements in predictions on how climate change will impact the dynamics of HABs. For a better and deeper understanding, the co-occurrence or interaction between climatic changes and temperature variations with other stressors are also summarized in this section. As climate change is considered as an important factor in HABs formation, associated changes such as increasing temperature and precipitation are of wide ranges and no certainties exist regarding their effects on flow and stratification [13]. Climatic change causes spatio-temporal variations in the hydrological aspects and rainfall patterns, which have increasing effects on the over-enrichment of nutrients modulating the HABs [48]. Therefore, the variability in patterns of rainfall significantly impacts sediment and nutrient delivery. Flushing, water residence time, metabolism, sediment-water exchange, and vertical stratification, may affect the persistence and dominance of HABs [12,49,50]. Some of the species such as rhodophytes and dinoflagellates increased rapidly while some are mostly dominating, for example, *Dinophysis acuminata*, *Prorocentrum minimum*, *Chattonella antiqua*, and *Fibrocapsa japonica* were favored by these factors in coastal zones of the North Sea [13].

The interaction between climatic change and temperature variations is directly correlated with the growth of phytoplankton, because their growth increased rapidly with increased temperature [51]. Numerous studies have reported the higher algal biomass in temperature ranges of 25–30°C [52]. However, variations in temperature affect the cyclic patterns of water that may change the physiological structure of water by elongating the stratification period in turn, which favors the HAB formation [11]. Similarly, global warming is associated with temperature changes, which significantly affect aquatic ecosystems. If the average value of temperature increases, this might have a direct effect on algal blooms. Globally, increased temperature up to 1.5–5 °C is direct evidence of increased bloom formation during this century [53]. At this rate, if the temperature reaches or exceeds 20 °C, it will have an inverse relation with eukaryotic phytoplankton whose growth will be stopped or decreased while giving a competitive benefit to cyanobacteria by increasing their growth [13]. On the other hand, relationships between temperature, growth rate, and toxin production are highly strain-specific and species-specific. Like temperature increases (up to 25 °C), generally, the growth of *Dinophysis* spp. is promoted [54]. For other toxin-producing species, inverse relationships between toxin production and growth rate have been described. Specifically, the growth of *Alexandrium* spp. increases with temperature in a strain-specific manner. Toxin content is generally greater among slower-growing cells maintained at lower temperatures [55,56]. Similarly, toxin content (per cell) of yessotoxin-producing dinoflagellates within the genera *Protoceratium* is greater within slowly dividing cells [57,58].

Drastic climate change and increasing temperature also cause an imbalance in photosynthetic activities and phosphorylation mechanisms. Atmospheric carbon dioxide was previously increased at a rate of 1% per year, while it reaches 3% annually now and up to 800 ppm is expected at the end of this century [59]. Due to the rise in CO_2_, the chemical composition of water will alter with declining pH and carbonate ions, and it is understandable that cyanobacteria are favored by low dissolved inorganic carbon [60]. Alternatively, it competes with eukaryotic algae under high pH and low carbon dioxide conditions [61]. Under high CO_2_ the toxin production including saxitoxins by several harmful algae (e.g., *Alexandrium* spp.) increases [62]. However, species-specific responses always do not follow the common trends. For example, growth and toxin production by *A. catenella* and *A. ostenfeldii* are expected to increase under high CO_2_ levels [63,64]. Furthermore, low pH may decrease the rate of cell division in some cyanobacteria species that promote bloom formation [65]. Strain-specific differences have been reported for *P. multiseries*, with some groups reporting an increase in growth and toxin production at low pH [66,67]. For harmful cyanobacteria, the responses to acidification are phenotype-specific. Some toxic strains lose their competitive advantages over non-toxic ones at low pH, and others become more competitive [68,69,70]. While some cyanobacteria decrease the rates of their cell division in response to low pH conditions [65,71,72]. However, laboratory and field studies demonstrated that other cyanobacteria respond to increase CO_2_ with increased cell division and carbon fixation, or both [73,74,75,76,77].

Climatic changes may also affect the salinity in estuaries and freshwater systems due to rising sea levels, an increase in drought frequency and duration in some regions and a concomitant increase in desiccation, or in other areas, increases in precipitation due to storms [78,79]. Although many phytoplankton species have no tolerance to salinity changes, however, some species, such as *Lyngbya* and *Trichodesmium* are able to survive in environments with euryhaline patterns [80]. Thus, changes in salinity may cause the shifts in phytoplankton communities and it may affect the composition of the community and potentially toxic concentrations [81]. Overall, warmer temperatures are beneficial for some HABs with expanded realized niche and accelerated growth [26,28,82,83,84]. A species-specific relationship exists between temperature, growth rate, and toxicity in some algal species. For example, the promoted growth and increased diarrhetic shellfish poisoning in *Dinophysis* spp. observed with increasing temperature up to 25 °C [43,54]. For some other toxin-producing species, there is an inverse relationship between growth and toxin production such as *Alexandrium* spp. which has increased growth at the optimum temperature of 15 °C, and increased toxin production at a higher temperature of 22 °C [55,56]. However, for other species, the relationship between temperature, growth, and toxin production is variable [85]. A number of studies investigated the impacts of warming/temperature on the growth rate and toxin profile of HABs. However, some of them have summarized the important interactions between marine and freshwater HABs with other stressors and toxin activity. In summary, the interaction between climatic patterns and other environmental stressors is a diverse phenomenon; critically, rainfall patterns may alter the population dynamics of HABs.

### 2.2. Nutrient Flooding and Eutrophication

Heisler et al. [5] in a scientific consensus on eutrophication and HABs revealed that degraded water quality from increased nutrient pollution promotes the development and persistence of many HABs. The composition, not just the total quantity of the nutrient pool affects the HABs, both chronic and episodic nutrient delivery promotes the HAB development [5]. O’Neil et al. [18] highlighted that eutrophication may promote proliferation and expansion of cHABs, particularly cyanobacterial genera from freshwater (*Microcystis*, *Anabaena*, *Cylindrospermopsis*), estuarine (*Nodularia*, *Aphanizomenon*), and marine ecosystems (*Lyngbya*, *Synechococcus*, *Trichodesmium*). Zohdi and Abbaspour [42] demonstrated that in eutrophic regions, the growth rate of HABs is maximum due to the entry of contaminated river flow and organic wastewater, which includes more than 10 ng/L of vitamin B12 concentration. Furthermore, among the potential drivers contributing to HAB events, the role of nutrient inputs has gained the most attention worldwide. Overpopulation of human beings enhanced the cultural nutrient loading which tracks their way and empties into the aquatic resources causing larger and fast bloom formation [5,29,86]. As freshwater bodies get enriched with nutrients, mainly phosphorus, a community shift may occur leading to the dominance of phytoplankton biomasses [18,82]. in the same way, phosphorus may promote akinete production in *Anabena* spp. as well, in limited nutrient conditions, P also favors the growth of *Microcystis* [18,87]. These changes are best exemplified by dense bloom formation in newly eutrophic rivers, lakes, and reservoirs that were previously devoid of cHABs [18]. For example, Pampulha reservoir in Brazil and Taihu lake in China faced dense and persistent cyanobacterial blooms [88,89].

Intensified anthropogenic interventions in the form of industrial waste and sewage disposal in aquatic ecosystems containing nitrates, ammonia, and phosphates also favor the HAB formation [90]. A higher quantity of phosphates in coastal water increased the ratio of nutrients that highly influenced the growth of harmful and nontoxic blooms. Some species are usually nontoxic but if they are exposed to different nutrient concentrations, they become toxic in cultural nutrient loading [91]. Evidence suggests that N may be equally or more important than P in the occurrence of toxic, non-diazotrophic cyanobacteria blooms such as *Microcystis*. Further, laboratory investigations demonstrated that increasing N concentrations generally increase the *Microcystis* growth and toxicity [18]. Thus, as nutrient loading increases the ratio of phosphorous and nitrogen to silicates, it favors the growth of harmful algal species including some non-diatoms. For example, in the Black Sea, silicate concentration decreases causing an escalation in blooms of flagellates and diatoms [11]. It is also believed that low nitrogen to phosphate ratios, or higher phosphate ratios could favor cyanobacterial blooms [92,93]. In addition, micronutrients such as trace metals also favor phytoplankton growth and have a critical role in the assimilation of essential macronutrients, photosynthesis, and toxicity of some algal species. The phenomenon of eutrophication produced HABs, which in turn produces harmful toxins and the activities of these toxins ultimately affect the vulnerability of aquatic organisms (Table 1).

**Table 1:** Summary of known interactions between marine and freshwater harmful algal blooms and other stressors.

Factors	Stressor	HABs Species	Strain	Toxic Mechanism	Test Organism	Effects or Observations	Reference
Climate changes	Thermal stress	*Microcystis aeruginosa*	UV-006	Microcystin	*Mus musculus* (Mouse)	Diminished toxicity at warmer temperatures	[94]
Climate changes	Thermal stress	*Microcystis aeruginosa*	M228	Microcystin	*Mus musculus* (Mouse)	Higher LD_50_ at a warmer temperature	[95]
Climate changes	Thermal stress	*Cochlodinium polykrikoides*	CP1	Reactive oxygen species (ROS)	*Argopecten irradians* (bay scallop)	Increased toxicity (i.e., inhibited swimming by larval scallops) at cold temperature	[96]
Climate changes	Thermal stress	*Cochlodinium polykrikoides*	CP1	Reactive oxygen species (ROS)	*Mercenaria mercenaria* (hard clam, northern quahog)	Increased lethality at cold temperature	[96]
Climate changes	Thermal stress	*Cochlodinium polykrikoides*	CP1, field samples	Reactive oxygen species (ROS)	*Menidia berrylina* (inland silverside)	Increased lethality at cold temperature	[96]
Climate changes	Thermal stress	*Microcystis aeruginosa*	CP1, CPSB-1 G	Microcystin	*Cyprinodon variegatus* (sheephead minnow)	Increased lethality at cold temperature	[96]
Climate changes	Thermal stress	*Microcystis aeruginosa*	Purified toxins-MCLR	Microcystin	*Danio rerio* (zebrafish)	Increased toxicity at warmer temperatures	[97]
Climate changes	Thermal stress	*Heterosigma akashiwo*	Purified toxins-MCLR	NA	*Moina macrocopa* (freshwater daphnids)	Increased toxicity at warmer temperatures	[98]
Eutrophication	Acidification	*Aureococcus anophagefferens*	CCMP 2393	Unidentified toxins	NA	Increased swimming speed and net-down movement of algal cells in high *p*CO_2_ environments	[99]
Eutrophication	Acidification	*Aureococcus anophagefferens*	CCMP 1984	Unidentified toxins	*Argopecten irradians*	Increased lethality in low pH treatments	[100]
Eutrophication	Acidification	*Cochlodinium polykrikoides*	CCMP 1984	Unidentified toxins	*Crassostrea virginica* (eastern oyster)	Increased lethality in low pH treatments	[100]
Eutrophication	Acidification	*Cochlodinium polykrikoides*	CP1	Reactive oxygen species (ROS)	*Mercenaria mercenaria*	Increased mortality by larvae in acidification treatments	[101]
Eutrophication	Acidification	*Microcystis aeruginosa*	CP1	Reactive oxygen species (ROS)	*Argopecten irradians*	Increased mortality by larvae in acidification treatments	[96]
Upwelling	Hypoxia	*Microcystis aeruginosa*	FACHB-905	Microcystin	*Hyriopsis cumingii* (sail mussel)	Reduced scope for growth among mussels within hypoxic treatments	[102]
Upwelling	Hypoxia	*Microcystis aeruginosa*	FACHB-905	Microcystin	*Hyriopsis cumingii* (sail mussel)	Diminished immune response among mussels within hypoxic treatments	[103]
Upwelling	Hypoxia	*Microcystis aeruginosa*	FACHB-905	Microcystin	*Hyriopsis cumingii* (sail mussel)	Increase cellular damage among mussels within hypoxic treatments	[104]
Upwelling	Hypoxia	*Stephanopyxis palmeriana*	NA	Unidentified toxins	NA	Increased seasonal toxicity	[105]

Organic fertilizers from animal farming flushed out into the seawater are the major contributors to eutrophication in coastal regions. Vertical nutrient mixing can form heavy biomass, causing anoxia and detriment to the ecosystem [11]. Flushing of rainwater into the sea also increases the nutrients and algal biomass, as reported in the regions of the Black Sea and the Caspian Sea. On the Texas coast in 1935, heavy rainfall caused excessive phytoplankton blooms [106,107]. Whereas, the unusual rainfall in other places also caused events of *L. polyedrum* blooms, e.g., in Santa Barbara and south of the Mexican border due to enriched coastal water [1]. Garcia-Hernandez et al. [108] reported that increased shrimp farming activity might drastically change the Rhaphidophytes, diatoms, and dinoflagellate blooms, which may occur because of heavy discharge of nutrients ultimately leading to the increased eutrophication that triggers algal bloom formation. Interactive effects of eutrophication/nutrient flooding and HABs are diverse and complex; however, much of the current knowledge provides insights into these processes which seems to enhance the frequency and magnitude of these events in the future.

### 2.3. Events of Upwelling

The vertical or upward movement that causes circulation and increases productivity in the photic zone, which in turn changes the environmental conditions and nutrient content is termed upwelling [10]. Upwelling zones are considered the world’s best productive areas, whereas some researchers reported that upwelling could halt the occurrence of red tide. The phenomenon of upwelling that occurred between 1998 and 2004 was associated with HAB events in Hong Kong during summer, which occurred in conjunction with Manson winds and river water runoff [106,107]. Some studies in India were conducted along Dakshina Kannada coast and reported the occurrence of upwelling from March to October in that region, which was associated with HABs on the southwest coast of India [105]. Kun Kaak Bay in the south of Sonora, Mexico and northwest of Kino Bay have high productivity suggesting the upwelling of currents, and in the past, the diatom blooming species (e.g., *Stephanopyxis palmeriana*) were more common blooms. Similarly, red tide nontoxic blooms are commonly found in the Gulf of California. *Mesodinium rubrum* is a more common red tide algal species that was also associated with upwelling in these regions [108]. Events of upwelling mostly influenced the mixing of water layers, producing an imbalance in the mixture of water and gases; particularly, hypoxic conditions lead to the development of blooms and might cause serious toxicity to organisms [42,102,103,104]. However, the events of upwelling interact with climatic changes. HAB dynamics have traditionally been challenging to quantify, however, continuous efforts can push through this emerging challenge.

### 2.4. Wind Pressure/Strength and Dust Storms

Wind can contribute to the upwelling of deeper water layers rich with nutrients, which enhances the opportunity for HAB formation. Similarly, dust storms rich with iron increased nutrients in the sea when blowing through large desert areas such as the Sahara. In east coastal regions of North America, iron is considered a contributing factor to red tides [106]. The University of South Florida reported in their satellite-based observations, that clouds of dust blowing over the Sahara Desert and across the Gulf of Mexico and the Atlantic Ocean are the major factors responsible for red tides and the water of West Florida is provided with iron from these giant clouds [109]. *Trichodesmium*, a cyanobacterial genus, utilizes this iron from dust to fix atmospheric N_2_ that makes the environment of the Gulf of Mexico productive for toxic algae forming the red tides [109]. Albert et al. [3] reported some factors responsible in coastal Queensland (Australia) for *Lyngbya majuscula* blooming. During rainfall, water carried phosphorous, iron, and other organic contaminants to the coastal region. The combination of iron and phosphorous is required for *L. majuscula* to enhance its photosynthetic activity which expands its population and forms larger blooms. Iron is considered an important source for nitrogen acquisition and when the iron is highly available there is a high chance of brown tides, for example, brown tide bloom produced by *Aureococcus anophagefferens* is associated with high iron availability. The events that occurred in the Peconic estuary, New York, USA, may be explained by these observations [110]. Additionally, higher concentrations of heavy metals such as Pd and Cd in untreated nutrient-rich effluents originating from aquacultural practices is also a good example of increasing metal toxicity in oceans leading to the formation of HABs [108].

### 2.5. Discharge of Ships/Ballast Waters

It is estimated that the ballast waters of ships’ tanks can transfer approximately 300 billion dinoflagellates [42]. In the Persian Gulf during 2008, the occurrence of a red tide was predicted to be caused by the ballast water of ships along the Qatar and Kuwait routes [111]. *Noctiluca* dinoflagellate growth has increased, causing red tides as a result of oil spills from ships [96]. Wastewater containing pathogens and nutrients discharged from ships results in increased eutrophication, algal growth, and a decrease in soluble oxygen in water [112]. Anil et al. [113] observed that the spreading of harmful algal species via ballast water is very limited; however, ballast water may serve as a vector for the dispersal of harmful algal bloom species. For example, *Monostroma oxyspermum*, an algal species, was introduced from the northwest pacific and northeast Atlantic to India’s west coast due to the discharge of ballast waters [114]. The bloom formation by shipping from other oceans increases the susceptibility of HABs; however, large-scale studies are required to explore the causes of fast-spreading HAB species [114].

### 2.6. Other Factors Involved

Photosynthetic characteristics of phytoplankton indicated that their growth required enough light penetration, light intensity, and day/night durations. Mostly, the arousal of red tides was observed during warm sunny periods, which indicated the importance of light factors for these tidal formations [42]. Among other factors, one prominent cause of red tide algal blooming is also favored by their lower consumption by consumers and their grazers [106]. Unhealthy corals also favor bloom formation because the deposition of sediments makes the corals unhealthy or dead, whereas other zooplankton and fish death in the food web later covers the crust with calcareous matter and algae leading to bloom formation [115]. Volcanos and earthquakes on the seabed cause heat generation due to earth layer friction and increase the possibility of dissolving materials such as iron and mineral compounds in water. For example, in 2010, iron-dissolved ash from the *Eyjafjallajökull* volcano created an algal bloom in the North Atlantic [42]. Collectively, whatever the causes or reasons, globally aquatic resources especially in the coastal regions are now subjected to an unprecedented variety and frequency of HAB events.

## 3. Impacts of Harmful Algal Blooms (HABs)

HABs have large ecological and socioeconomic costs, affecting agriculture, real estate, food web resilience, water quality, fisheries, drinking water, tourism, and habitats, contributing to fish death and anoxia [116]. These effects can result in fish death involving thousands of fish and other marine life, leading to the degradation of the ecosystem [117]. Major impacts of HABs are summarized in the sections below.

### 3.1. Shellfish Poisoning

When toxic blooms are filtered by shellfish as their food source, the shellfish accumulate toxins in them up to dangerous levels for humans and other consumers. These toxicity events lead to poisoning syndromes such as amnesic shellfish poisoning (ASP), neurotoxic shellfish poisoning (NSP), paralytic shellfish poisoning (PSP), Ciguatera fish poisoning (CFP), diarrhetic shellfish poisoning (DSP), and cyanobacterial toxin poisoning. Besides these, there are many species that cause water discoloration, some are non-toxic to humans but lethal for invertebrates. Marine microalgae are important food for the inhabiting fauna from their early life stages, particularly mussels, clams, oysters, scallops, finfish, protozoans, and crustaceans. However, more than 5000 species of these microalgae have been identified [117], and out of these, only 300 contribute to the formation of red tides in marine ecosystems [118].

More than 50 species or so have been identified to produce toxins and cause human physiological mechanisms by fish or shellfish poisoning. Globally important harmful algal bloom species that cause paralytic shellfish poisoning (PSP) are *Alexandrium catenella*, *A. cohorticula*, *A. fundyense*, *A. fraterculus*, *A. minutum*, *A. tamarense*, *Gymnodinium catenatum*, *Pyrodinium bahamense*, and *Lyngbya* spp. Some calcareous macroalgae; those that cause diarrhetic shellfish poisoning (DSP) include: *Dinophysis acuta*, *D. acuminate*, *D. caudata*, *D. fortii*, *D. norvegica*, *D. mitra*, *D. rotundata*, *D. sacculus*, and *Prorocentrum lima*. Amnesic shellfish poisoning (ASP); *Pseudo-nitzschia australis*, *P. delicatissima*, *P. multiseries*, *P. pseudodelicatissima*, *P. pungens*, and *P. seriata*. Ciguatera fish poisoning (CFP): *Gambierdiscus toxicus*, *Coolia monotis*, and *Fukuyoa* spp. Neurotoxic shellfish poisoning (NSP): *Karenia brevis*, *K. papilionacea*, *K. selliformis*, and *K. bicuneiformis*. Cyanobacterial toxin poisoning: *Anabaena* *circinalis*, *Microcystis aeruginosa*, *Nodularia spumigena*, *Pfiesteria piscicida*, and *P. shumwayae*. Those producing harmless water discolorations are: *Akashiwo sanguinea*, *Gonyaulax polygramma*, *Noctiluca scintillans*, *Scrippsiella trochoidea*, and *Trichodesmium erythraeum*. While, some notorious species which are harmful to invertebrates and fish, but non-toxic to humans include: *Chaetoceros concavicorne*, *C. convolutes*, *Karenia mikimotoi*, *K. brevisulcata*, *Karlodinium micrum*, *Chrysochromulina polylepis*, *Prymnesium parvum*, *P. patelliferum*, *Heterosigma akashiwo*, *Chattonella antiqua*, *C. marina*, and *C. verruculosa* [1,118,119].

### 3.2. Human Health Impacts and Toxin Production by HABs

Marine toxin diseases are categorized based on their trans-vector types. For example, shellfish carry toxins that lead to paralytic shellfish poisoning. Paralytic shellfish poisoning is caused by the shellfish that carried potential toxins of the disease. Whereas, poisoning through mollusks also occurred during algal bloom episodes. However, fish poisoning is regional and usually associated with fish or specific reefs. Bloom episodes of *Pfiesteria* dinoflagellate in estuaries of the Southern Atlantic coast and middle suggest that anthropogenic pressure on the aquatic environment aggravated the existing conditions. Consequently, this anthropogenic stress resulted in fish death and human health hazards [120]. Additionally, humans could be exposed to the toxins directly released into the air or water. This phenomenon occurs naturally or through human activities such as water treatment causing turbulence in the water and leading to the direct release of toxins by cell disruption. Toxins can enter the body through inhalation, causing associated respiratory symptoms such as irritation, coughing, and other ailments [117]. However, the phenomenon of algal toxicity and the interaction of human health hazards to this toxicity is very diverse and complex. Some major categories of algal toxins more commonly causing human health concerns are summarized in this section.

Furthermore, the production of phycotoxins changes with geographical location, and novel toxins are detected with spatial variation leading to serious toxic events. Variations in temporal patterns and fluctuations in environmental factors also influence the rates of toxin production within blooms [12,28,121,122]. Phycotoxins including cyanotoxins produced by cyanobacteria, and marine biotoxins produced by various harmful algal blooms species, can be lethal to a variety of organisms and human beings [123,124]. Some of the important phycotoxins are summarized in Table 2.

### 3.3. Ecological and Economic Impacts of HABs

The complex phenomenon of chemical signaling from HAB species can make it more complicated and difficult to comprehend the important and variable effects of HABs on organisms and ecosystems. HAB species have a variety of poisonous, noxious, or allelochemical qualities that allow them to escape from some effects and predation [125]. Micro-zooplankton and meso-zooplankton members could not consume the toxic HAB species often; however, these deterrent properties may be highly species-specific or condition-specific [33,126,127]. Toxins and allelochemicals of HABs may have potential impacts on the communities of organisms elsewhere in the food chain or food web, such as microbial food web alteration, especially when grazing pressures are low [128]. Such large-scale ecological problems of HABs have been notoriously difficult to evaluate and quantify.

The economic effects of HABs are variable and highly diversified, and the accurate costs of HABs are difficult to assess. However, some studies reported their conclusions, for example, Hoagland et al. [129] estimated the annual economic effects of HABs in the USA between 1987 and 1992. At that time, the economic effects were valued at USD 50.0 million per year. Anderson et al. [130] reported approximately the effect of USD 50 million on the US economy per year. These losses were mostly due to the commercial fisheries and human health impacts on marine ecosystems. Whereas, in freshwater ecosystems estimated losses in the US were USD 4.6 billion per year, related to the potential eutrophication [131]. Most of the studies have focused on the events of HABs in the US; however, exact costs are still difficult to assess [132]. Few other exceptions also exist. In Canada, the human health costs were (670,000 CAD/year), in the UK up to 118,00 GBP/year due to the impacts on commercial fisheries [133], whereas in other European countries the costs for monitoring and management range from EUR 30,000 to over EUR 7 billion/year [134,135]. In Australia, the estimated cost was AUD 1–8.7 million AUD/year [136], and in New Zealand approximately 50,000/quarter NZD [33].

The dire need to predict and minimize the above-mentioned HAB incidents offers a powerful impetus for the investment in research and management measures. Furthermore, Bernard et al. [137] assessed that the annual economic losses by HABs in freshwater and marine ecosystems as of 2014 could be at USD ±10 billion. However, based on this calculation and using the typical value of information (VOI), the estimate of the resource is 1% [138]. Whereas, a comprehensive system for detecting and forecasting the HAB information would represent the value of USD ±100 million annually. This would be the first and most reasonable estimate for the assumption of “how much and how should” be capitalized in the monitoring efforts for HABs [33].

### 3.4. Other Negative Impacts of HABs

#### 3.4.1. Effects on Aquaculture Food Production and Water Supply

HABs have negative effects on aquaculture and shellfish aquaculture because the shellfish have the ability to accumulate phycotoxins in their body by filtering during feeding, which also impacts their survival, life, history, and body structure [119,139]. Despite the large geographical distribution and negative impacts of HABs on aquaculture, accurate calculations for the estimated loss are difficult; however, some studies rigorously provided insights on this issue. According to FAO (2006), certain HABs constitute serious threats to aquaculture food production, that are linked to decreasing wild fish stocks, which have become a major protein source for coastal human populations [140]. The most effective strategy to protect humans from seafood poisoning related to HABs in farming and wild harvest of shellfish is to monitor the HAB species and biotoxins to implement the periodic closures of recreational, harvesting, growing, or commercial areas [141]. Moreover, the contamination of seafood items and products can result in financial losses in shellfish collection and cultivation, as well as certain finfish aquaculture. Besides these, other losses in auxiliary industries may be the distribution, processing, retailing, and wholesaling of seafood [142,143].

Zohdi and Abbaspour [140] found that HABs discolor coastal waters and harm the water quality. This issue results in damaging desalination systems and equipment, ships, and shore facilities. For instance, the Toledo water treatment plant could not treat the water to a safe level in 2014, so authorities gave hints to its 500,000 residents not to drink tap water. Kenya in 2004, China in 2007, and Australia in 2016 experienced similar incidents.

#### 3.4.2. Effects on Other Organisms

Mostly the presence of HABs is associated with large mortalities of fish, sea turtles, seabirds, mammals, and marine organisms. The whole cycle of toxin transfer can be considered as HABs release toxins that can be accumulated by oysters (shellfish), accumulating the toxin in their body and transferring it to other fish that consume oysters as food. Furthermore, as the food web continues these toxins enter human bodies by consuming seafood; however, this phenomenon can cause economic loss and also severe illness. Paradigms of ecological risk that attempt to determine the toxic effects of harmful algal blooms on the coastal ecosystem are actually analogous to, and also evolved from, the human health risk assessment context [144]. HABs also cause acute/chronic effects in mammals and other organisms including humans worldwide by producing harmful toxins [116]. Moreover, the toxin production of harmful algal blooms such as *Alexandrium tamarense* and *Gymnodinium catenatum* at high concentrations decreased the growth of protozoan communities [145]. Naturally occurring toxins within the HABs have the potential to kill fish, shellfish, and other microorganisms directly, or they may be retained in the bodies of these organisms and later could be transferred to the food webs [146,147]. Currently, ecological risk assessment has some limitations. Human health risk assessments posed by toxin production associated with algal species are definite but their mode of action and their respective classes are still speculative. Interacting populations of many algal species vary according to the spatio-temporal patterns and their toxin profile is also influenced by location and time duration [144]. However, we summarized the important harmful phycotoxins classes with clinical implications on humans.

### 3.5. Grazing Defense by HABs

#### 3.5.1. Defense of Phytoplankton against Grazers

During favorable nutrient loading conditions, the phytoplankton biomass increases. If certain factors depress the abundance of zooplankton the balance between phytoplankton and zooplankton biomasses is disturbed; then, phytoplanktons ultimately increase their population and cause blooms. Consequently, it is difficult for grazers to graze the density of these blooms on a normal ratio. As a result, some density-dependent toxins and extracellular polysaccharides might be present in the blooms causing harmful effects on the grazers. The best example of such a defense is shown by *Aureococcus anophagefferens* and *Aureoumbra lagunensis* during the 1985 and 1990–1997 blooms in Narragansett Bay at low grazer abundance periods [7,148]. This phenomenon makes it risky for the grazers to feed on the toxic phytoplankton at higher biomass and they show less grazing response to these toxic species [8,149]. However, if the algal population is low or normal, the growth rate of protozoans increases with an increase in food density until it reaches a specific maximum level [150,151,152].

Feeding by zooplanktons on harmful algal species also puts them into the category of HAB predators [153,154,155,156]. The population density of microzooplankton and their prey phytoplankton have equivalent growth rates [157]. Therefore, it is possible that grazing on HABs might result in optimal reproduction and growth in protozoan communities [158,159]. However, some grazers including protozoans and copepods successfully graze on some toxic dinoflagellates such as *Alexandrium, Gymnodinium*, *Prorocentrum minimum*, or *Heterocapsa circularisquama* without any apparent harm [156,158,159]. In a recent bioassay experiment on two harmful algal blooms, *Alexandrium tamarense* and *Gymnodinium catenatum*, increased feeding of periphytic ciliates on these blooms up to the algal concentrations of 10^2^ to 10^4^ cells mL^−1^ was reported [145]. Additionally, *Favella ehrenbergii* is a protozoan considered a voracious grazer of (*Alexandrium tamarense*) blooms [160].

During blooms, an increase in phytoplankton biomass will lead to an increase in respiration rates and total oxygen demand by both autotrophs and heterotrophs and this may lead to hypoxic conditions. In eutrophic bays, estuaries, and coastal lagoons, high photosynthetic activity and heavy phytoplankton biomass may lead to an increased pH and reduction in CO_2_ in marine water. Under highly eutrophic conditions, the grazers face different exposure circumstances of low oxygen at night and high pH during the daytime [161]. On the other hand, the hypoxic conditions are lethal for copepods causing increased mortality and reduced reproduction rates in female copepods [162,163]. Whereas, both hypoxic and anoxic conditions affect the distribution of planktonic protozoa. Some species are well adapted for anoxic conditions, some can flourish in normoxic environments, and others can dominate in hypoxic conditions [164]. An example is the Texas Laguna Madre dominated by the uninterrupted *Aureoumbra lagunesis* blooms. The density of *A. lagunesis* was higher and inversely proportional when compared to the protozoan’s grazer densities [165]. One possible reason for the difficulty of protozoa to feed and grow on high densities of *A. lagunensis* cells is the thick polysaccharide layer that surrounds cells, referred to as the extracellular polymeric substance or EPS [166]. Another reason for the suppression of natural populations of microzooplankton that feed on HABs depends on the size range of algal species [164]. For example, the cell diameter of *A. laguensis* is 4–5 μm, which does not fall in the preferred size ranges of the dominant copepod in Laguna Madre [167]. Moreover, the other suppression in the zooplankton population under the bloom condition could have been due to the high pH and hypoxic conditions. Based on these results, it seems that the initial grazer disruption, along with the nutrient pulse that allowed for rapid initial growth, was essential for the bloom to reach high densities of up to several million cells mL^−1^ within a few months [161]. Once this density of algal biomass reached a very high level, the planktonic grazers were suppressed for the duration of the *A. laguensis* bloom [165]. However, on the other hand, the algal defense theory suggested that a decrease in the density of edible algae by overgrazing leads to its replacement by slow-growing resistant species [168,169]. A study by McCauley and Briand is supporting the idea that the number of resistant species can be controlled by a reduction in grazing. The resistant species are defined as algae larger than 50 μm and almost all are cyanobacteria. However, low grazing conditions may be favoring some resistant species, for example, the Synedra a needle-like, unicellular diatom [169,170]. Therefore, this assumption would not contradict the fact that an increasing population of diverse nature of harmful algae leads to a higher resistance towards their predators, posing a serious threat to the aquatic environments.

#### 3.5.2. Threshold Effects of HABs on Grazers

Disruption in grazer populations (top to down controls) can be a contributing factor to phytoplankton blooms. The imbalance in zooplankton–phytoplankton interactions or if there is a lag between zooplankton populations and growth of phytoplankton, or some other factor depresses the abundance of potential grazers, phytoplankton populations may be temporarily released from grazer control and reach bloom densities [161]. When HABs reach higher densities, threshold levels also reach maximum levels, for example, the initiation of the brown tide bloom *Aureoumbra lagunensis* in Texas during 1985 and the Rhode Island bloom of *Aureococcus anophagefferens* in Narragansett Bay, arising during the low grazer abundance periods [7]. Once these blooms have originated, the zooplankton show reduced grazing often on these unpalatable species eventually leading to the extension and persistence of HABs [8]. This decrease in zooplankton grazing may impair nutrient regeneration, resulting in phytoplankton nutrient limitation [171]. Reduction or limitation in nutrient supply or altered nutrient ratios could be a potential increase in the toxicity of HAB species [172]. Although it has yet been demonstrated, it could be interesting to determine if anything analogous to quorum sensing in bacteria in toxic phytoplankton, perhaps the high-density blooms, could release chemicals that induce the additional production of toxins [161].

**Table 2:** The scientific consensus on phycotoxins by harmful algal blooms (HABs), the toxicity mechanism, diagnosis, and negative effects on human health.

Biological Category	Types	Toxicity Mechanism	Diagnosis	Symptoms	Reference
**Hapatotoxins**	Microcystin MCs	Carcinogenesis, genotoxicity, inhibition of protein phosphatases, repeated low-level exposure	Exposure; drinking water, contaminated dialysis fluid, soft water recreational environments	Liver hemorrhage, diarrhea, abdominal pain, vomiting, shock, jaundice, dyspnea, weakness, multiple organ failure, respiratory distress	[173]
	Nodularin	Inhibition of protein phosphatases	Drinking water, recreation	Goose bumps, diarrhea, liver hemorrhage, vomiting, weakness	[123]
	Cylindrospermopsin	Glutathione and protein synthesis as well as cytochrome P450. Repeated low level exposure; carcinogenesis, genotoxicity	Chronic exposure linked to cancer	Gastroenteritis abdominal pain, bloody diarrhea, vomiting, acute liver inflammation. Liver and kidney failure, asthma, hay fever	[123]
**Neurotoxins**	Anatoxin-a/Homoanatoxin-a	Nicotinic receptors: irreversible link to the nicotinic receiver S of acetylcholine in neuromuscular junction	Could be lethal	Muscle twitching, staggering, cramping, convulsions, paralysis, respiratory failure, gasping, death by suffocation	[116]
	Anatoxin-a (S)	Irreversible inhibitor acetylcholinesterase		Muscle twitching, salivation, paralysis, cramping	[174]
	Saxitoxins	Neurotoxic, target the peripheral nervous system. Selective high affinity block sodium conductance in voltage-gated sodium channels	Death can occur within 2–12 h after exposure. Good prognosis after 24 h, requiring good medical support system	Nausea, perioral burning ataxia, vomiting, drowsiness, muscular paralysis, paraesthesia, tachycardia, fever, respiratory failure, death	[175]
	beta-Methylamino L-alanine (BMAA)	Experimentally acts predominantly on motor neuron-excitotoxic through glutamate receptors	Chronic exposure linked to chronic neurodegenerative conditions: Amyotrophic Lateral Sclerosis	Not fully elucidated. Implicated in chronic neurodegenerative diseases	[40]
**Dermatoxins**	Aplysiatoxins	Potent tumor promoters Potentiation of protein kinase C		Asthma-like symptoms, skin irritation	[176]
	Lyngbyatoxin	Potent tumor promoters Potentiation of protein kinase C		Skin irritation, contraction in smooth muscles	[177]
**Biotoxins [Amnesic]**	Domoic acid and isomers	Production of excessive gastric juice increased the acidity	Consumption of shellfish (possibly, fish)	Diarrhoea, nausea, vomiting, dizziness, headache, confusion, short-term memory deficits, motor weakness, disorientation. Severe cases result in cardiac arrhythmia, seizures, coma, respiratory distress, and possibly death	[178,179]
	Domoic acid	Gastroentritis and neurotoxic	Consumption of infected clams oysters, crabs, anchovies, and sardines	Gastroenteritis, nausea, diarrhoea, vomiting, abdominal cramps within 24 h. Neurological symptoms such as headache, dizziness, respiratory problems, seizures, short-term memory loss and coma usually appear within 48 h	[10,180]
**[Azaspiracid]**	Azaspiracid and its derivatives	NA	Consumption of shellfish	Diarrhoea, nausea, vomiting, severe abdominal cramps; effects on mice include severe damage to the intestine, spleen, and liver tissues in animal tests	[181,182,183]
**[Ciguatera]**	Ciguatoxin	Gastrointestinal acidification	Consumption of coral reef fish	Nausea, diarrhoea, vomiting, numbness of mouth and extremities. Neurological symptoms may persist for several months	[184,185]
	Ciguatoxins/maitotoxin	NA	Consumption of small-algae eating fish	Paresthesias, pain in urination, pain in the teeth, temperature reversal, blurred vision, gastrointestinal effects; diarrhoea, vomiting, abdominal cramps. Cardio-vascular symptoms; arrhythmias and heart block	[10,180]
**[Diarrhetic]**	Dinophysistoxins	NA	Consumption of shellfish	Abdominal cramps, nausea, severe diarrhoea, vomiting, respiratory distress	[186]
	Okaidic acid	NA	Transferable through mussels, scallops, clam	Nausea, diarrhoea, abdominal cramps, vomiting, and chills within 30 min to 12 h of ingestion	[1]
**[Neurotoxic]**	Brevetoxins	Suppress the functioning of the nervous system slowly	Consumption of shellfish (and fish at least for marine mammals); inhalation of marine aerosols during active blooms	Temperature sensation, nausea, muscle weakness, reversals, and vertigo. Exposure to aerosols related to respiratory and eye irritation, particularly for asthmatics	[187,188]
**[Palytoxicosis]**	Palytoxin, Ostreocin, Ovatotoxin	NA	Cosumption of seafood; inhalation of marine aerosols; direct contact with water	Nausea, abdominal cramps, vomiting, severe diarrhoea, lethargy, tingling of the lips, mouth, face and neck, lowered heart rate, skeletal muscle breakdown, muscle spasms and pain, lack of sensation, myalgia and weakness, hypersalivation, and difficulty breathing. Exposure to aerosols; eye and nose irritation, rhinorrhoea, general malaise, fever. Cutaneous irritations in beach swimmers	[189,190]
**[Paralytic]**	Saxitoxin and derivatives	NA	Consumption of shellfish, crustaceans, fish	Diarrhoea, vomiting, nausea, numbness and tingling of the lips, mouth, face and neck. Severe cases can result in paralysis of the muscles of the chest and abdomen leading to death	[191]

## 4. Management of Harmful Algal Blooms (HABs)

The diversity and impacts of harmful algal blooms are an increasing challenge for their management. Although various efforts have been directed towards managing HABs, nevertheless, strategies are needed to protect the fisheries sector, minimize economic losses as well as ecosystem deterioration, and foremost protect public health; these have varied considerably on large spatial scales, and among HAB types. Anderson [192] highlighted strategies used by various countries and commercial sectors across the world for the monitoring and management of HABs in coastline waters. A few strategies have been discussed in the sections below.

### 4.1. Current Trends

Over the last three decades, the nature of the HAB problem has changed considerably across the world. In recent years, a dramatic expansion has occurred in the areas affected by PSP toxins. However, a similar pattern also applies to various other types of HABs. Events of toxic blooms, resources affected, economic losses by them, a variety of toxins, and the emergence of toxic species have increased all over the world [192,193,194]. However, the sole point of contention is about the motives for the growth of HABs [192,195]. Mostly it is immediately assumed that anthropogenic activities and pollution are involved, and in some cases, this is true [194]. A variety of HAB species flourish on nitrogen and phosphorus present in industrial effluents, sewage, and agriculture waste. However, closer examination has revealed that some extended or newly born HAB issues have arisen in the waters where pollution is not the main influence. For thousands, if not millions of years, HAB species have been on the planet. During this extensive time period, they have had sufficient opportunities to expand, aided by climate change, movement in tectonic plates, and other global scenarios. As a result of improved detection technologies and more observers, certain new bloom episodes may reflect indigenous populations that have been detected [192].

The anthropogenic intervention has also aided the global growth of HAB by carrying hazardous species in ship ballast water [195]. Extensive aquaculture practices in many countries are another factor driving the global expansion of HABs. This leads to better product quality and safety monitoring, uncovering indigenous hazardous algae that were most likely always there [193]. Anderson (2009) also highlighted that the construction of aquaculture facilities has placed fish and shellfish resources in areas where toxic algal species occur but were previously unknown, leading to mortality events or toxicity outbreaks that would not have been noticed had the aquaculture facility not been placed there. It is now clear that the global spread of HAB phenomena is due in part to our ability to better identify the problem’s boundaries, the nature and breadth of poisonous or hazardous species, as well as their consequences. However, HABs are a major and widespread problem that is far larger than previously realized [192].

### 4.2. Management Issues of HABs

Those in charge of managing coastal resources endangered by HABs have a huge challenge because of the range of blooms and their effects are diverse in nature. The solutions required to protect the fisheries sectors, limit the economic losses, and environmental deterioration, and protect public health vary greatly depending on where you are and what form of HAB you have [192]. Various strategies have come across and been adopted by different countries and several of them are discussed here.

### 4.3. Mitigation of HABs

Enormous management actions taken against the distribution and expansion of HABs can be termed as mitigation. These actions include dealing with the prevailing or current bloom, and necessary steps taken to reduce the possible negative impacts [192]. Routine monitoring programs, which are now being carried out in more than 50 countries, are aimed at detecting harmful levels of the HAB toxins in shellfish [192]. These types of studies will lead to harvesting restrictions to keep the contaminated products off the market. Another common mitigation strategy is dragging away the fish net pens from intense HAB sites [192]. Various other traditional approaches including a reduction in nutrient load and experimental methods (e.g., omnivorous fish removal, flushing, and artificial mixing) have been used to reduce the expansion of HABs [196]. Furthermore, recently Paerl et al. [196] reviewed several mitigation strategies for cyanobacterial HABs (cHABs) including their control within water and airsheds and within water bodies. Although a wide range of measures has been employed to deal with HABs, however, there is still little information available on how climate change and temporal patterns will influence the efficacy of all strategies within water bodies.

### 4.4. Prevention of HABs

Steps or actions performed to prevent HABs from occurring or having a direct impact on a certain resource can be summed up as prevention [192]. Increased use of chemical fertilizers in agriculture, as well as increased combustion of fossil fuels, have all contributed to a rapid increase in the inflow of plant nutrients, notably nitrogen compounds, into the coastal seas around the world [86]. Controlling sewage or waste disposal has the potential to avert certain types of HABs, as demonstrated by legislation or policy measures adopted in Seto Inland Sea and other sites [197]. Several countries are executing sewage reduction plans, which is a positive trend that should be supported [192].

### 4.5. Control of HABs

Harmful algal blooms can be controlled by wise management and the best time to control these entities is at their initial growth before their development. Apart from this, many other preventive measures can be used to avoid them, such as preventing the direct disposal of sewage and animal waste to the aquatic resources, proper handling of wastewater treatment, and establishing an equipped refinery to control the nitrogen-to-phosphorus ratios, the rectification of agricultural methods, utilization of fertilizers at proper times to decrease potential runoff, the establishment of barrier areas of wetlands and bush to ensure maximum removal of phosphorus before release to the water bodies, and application of conservation tillage to decrease runoff for preventing the HABs. Other detailed physical, chemical, and biological methods are discussed in the sections below and summarized in Table 3.

**Table 3:** Summary of methods for controlling HABs.

Control Measure	Target Algal Species	Action/Mechanism	Reference
**Physical Control**			
Hypolimnetic withdrawl and horizontal flushing	Dinoflagellates and Cyanobacterial species particualry (*Microcystis* and *Dolichospermum*)	Mechanical pumps, pneumatic or hydraulic mixtures are used to produce water mixture which improves water quality and avoids stratification.	[198,199,200]
Flocculation	Cyanobacterial blooms	Through adhesion and repeated collision, large, rapidly sinking aggregates (or flocs) of algae and clay are formed and settle on the ocean floor.	[201,202]
Sediment resuspension, burial, and removal	All bloom-forming species (dinoflagellates, cyanobacteria)	To resuspend sediments in an area thought to be a seedbed for algal cysts (thick-walled dormant cells of algae) with the objective of burying cysts in deeper oxygen-depleted sediments where they are unable to germinate and, to resuspend sediments that would act as a natural flocculant to remove algal cells from the water column. Burial can be achieved by the placement of offsite material over the treatment area. All offsite material would be clean and free of toxins and of similar grain size and composition to sediments of the treatment area. Burial is also achievable through hydraulic suction dredging, where dredged material is removed from one area and discharged over the treatment area.	[202]
Aeration	All bloom-forming species (dinoflagellates, cyanobacteria)	Aerators operate by pumping air through a diffuser near the bottom of a water body, resulting in the formation of plumes that rise to the surface and create vertical circulation cells as they propagate outwards from the aerator. This mixing of the water column disrupts the behavior of algal cells to migrate vertically in addition to limiting the accessibility of nutrients by internal loading.	[203]
Hydrologic manipulations	All bloom-forming species (dinoflagellates, cyanobacteria)	Manipulation of inflow/outflow of water in the system to disrupt stratification and control HABs.	[203]
Mechanical mixing (circulation)	Cyanobacterial blooms	Mechanical mixers are usually surface-mounted to disrupt the algal growth to migrate vertically in addition to limiting the accessibility of nutrients.	[203]
Reservoir drawdown/desiccation	Cyanobacterial blooms	Reservoirs and other controlled waterbodies can draw down the water level to the point where algal accumulations are exposed above the waterline. Subsequent desiccation and/or scraping to remove the layer of algal blooms attached to sediment or rock is required, in addition to the reinjection of water into the system.	[203]
Surface skimming	Cyanobacterial blooms	Oil-spill skimmers have been used to remove cyanobacterial bloom surface scums. This technique is often coupled with the implementation of some coagulant or flocculent.	[203]
Ultrasound	Cyanobacterial blooms	An ultrasound device is used to control HABs by emitting ultrasonic waves of a particular frequency such that the cellular structure of algal species is destroyed by rupturing internal gas vesicles used for buoyancy control.	[203]
**Chemical Control**			
Algaecides/Algaestats applied prior to bloom to resist bloom formation	Cyanobacterial blooms	Algaecides are chemical compounds applied to a waterbody to kill cyanobacteria and destroy the bloom. Several examples are copper-based algaecides (copper sulphate, copper II alkanolamine, copper citrate, etc.), potassium permanganate, chlorine, lime.	[203]
Biosurfactants	Species specific (depends on specific bacteria or yeast used to produce surfactants)	Surfactants break down algal cell membranes, making them non-functional, often resulting in cell lysis.	[202]
Barley straw	Cyanobacterial blooms	Barley straw bales are deployed around the perimeter of the waterbody. Barley straw, when exposed to sunlight and in the presence of oxygen, produces a chemical that inhibits algae growth. Field studies suggest significant algistatic effects. Several causes for the observed effects have been suggested; however, the exact mechanism of this process is not well understood.	[203]
Coagulation	Cyanobacterial blooms	Coagulants are used to facilitate the sedimentation of cyanobacteria cells to the anoxic bottom layer of the water column or below the photic zone. Unable to access light, oxygen, and other critical resources, the cells do not continue to multiply and eventually die.	[203]
**Biological Control**			
**Bacteria as Bio Controllers**			
*Bacillus cereus*	*Microcystis aeruginosa*	Secretion of cyanobacteriolytic substances	[204]
*Bacillus* sp.	*Aphanizomenon flos-aquae*	Cell-to-cell contact mechanism	[205]
*Bacillus* sp.	*Microcystis aeruginosa*	Production of an extracellular product	[206]
*Bacillus* sp.	*Phaeocystis globosa*	Secretion of algalytic substance	[207]
*Bdellovibrio*-like sp.	*Microcystis aeruginosa* (lake)	Penetration	[208]
*Brachybacterium* sp.	*Alexandrium catenella*	Produce secondary metabolites	[209]
*Cytophaga*	*Microcystis aeruginosa*	Direct contact	[210]
*Flexibacter flexilis, F. sancti*	*Oscillatoria williamsii*	Inhibition of the photosynthetic electron transport reactions, and glycolate dehydrogenase and nitrogenase activity	[211]
*Myxococcus fulvus* BGO2	*Phormidium lucidum*	Entrapment	[212]
*Myxococcus xanthus* PCO2	NA	Entrapment	[213]
*Pseudomonas fluorescens*	*Heterosigma akashiwo*	Indirect attack by alga-lytic substances	[214]
*Pseudomonas putida*	*Microcystis aeruginosa*	Inhibit the synthesis of the photosynthetic apparatus.	[215]
*Pedobacter* sp. MaI11-5	*Microcystis aeruginosa*	Mucous-like secretion from cyanobacteria for self-defense	[216]
*Raoultella* sp. R11	NA	Dissolved microbial metabolites and humic acid	[217]
*Rhodobacteraceae* PD-2	*Prorocentrum donghaiense*	Produce N-acyl-homoserine lactone signals	[218]
*Streptomyces neyagawaensis*	*Microcystis aeruginosa*	Secretion of extracellular antialgal substances	[219]
*Saprospira albida*	*Microcystis aeruginosa*	Parasitic lysis	[220]
*Streptomyces* sp.	*Microcystis aeruginosa*	Indirect attack by producing algicidal compounds	[221]
*Agrobacterium vitis*	*Microcystis aeruginosa*	Quorum sensing	[222]
*Rhizobium* sp.	*Microcystis aeruginosa*	Lysis	[223]
*Methylobacterium zatmanii*, *Sandaracinobactor sibiricus*, *Halobacillus* sp.	*Microcystis aeruginosa*	Bioflocculation	[224]
Algicidal bacteria (*Pseudoalteromonas* sp.)	*Chattonella* sp., *Gymnodinium* sp., *Heterosigma* sp.	Indirect/Algicidal effect	[225]
Algicidal bacteria (*Bacillus* sp. LP10)	*Phuphania globosa*	Indirect/Active compounds lytic	[226]
Algicidal bacteria (*Vibrio* sp., *Flavobacterium* sp., *Pseudoaltero* sp., *Acinetobacter* sp.)	*Gymnodinium mikimotoi*	Direct/Growth inhibition	[227]
Algicidal bacteria (*Bacillus* sp. AB-4)	*Chattonella marina*, *Akashiwo sanguinea*, *Fibrocapsa japonica*, *Heterosigma akashiwo*, *Scrippsiella trochoidea*	Indirect/Algicidal effect	[228]
Algicidal bacteria (*Vibrio* sp. DHQ25)	*Alexandrium tamarense*	Indirect/Algicidal effect	[229]
Algicidal bacteria (*Pseudoalteromonas* sp., *Zobellia* sp., *Cellulophaga lytica*, *Planomicrobium* sp., *Bacillus cereus*)	*Gymnodinium catenatum*	Indirect/Active compounds lytic	[230]
Biosurfactant bacteria (*Pseudomonas aeruginosa*)	*Alexandrium minutum*, *Karenia brevis*, *Pseudo-nitzschia* sp., *Gonyostomum semen*, *Microcystis aeruginosa*	Indirect/Surfactant	[231]
Algicidal bacteria (*Shewanella* sp. IRI-160)	*Prorocentrum piscicida*, *Prorocentrum minimum*, *Gyrodinium uncatenum*	Indirect/Algicidal effect	[232]
**Zooplankton as biocontroller**			
*Daphnia ambigua*	*Microcystis aeruginosa*	Grazing	[233]
*Daphnia hyaline*	*Chlorella* sp.	Grazing	[234]
*D. galeata, Cyclops* sp.	*Scenedesmus* sp.	Grazing	[234]
*Poterioochromonas* sp.	*Microcystis aeruginosa*	Grazing	[235]
*Strombidinopsis jeokjo*	*Cochlodinium polykrikoides*	Direct/Grazing	[236]
*Favella taraikaensi*, *F. azotica*	*Alexandrium tamarense*	Direct/Grazing	[237]
**Algae as bio controller**			
Dinoflagellate heterotrophic (*Stoeckeria algicida*)	*Heterosigma akashiwo*	Direct/Grazing	[238]
*Ankistrodesmus falcatus*	*Chlorella vulgaris*	Bio-flocculation	[239]
*Scenedesmus obliquus*	*Chlorella vulgaris*	Bio-flocculation	[239]
*Tetraselmis suecica*	*Neochloris oleoabundans*	Bio-flocculation	[239]
**Fungi as bio controller**			
Fungus *Trichaptum abietinum*	*Microcystis aeruginosa*, *M. flos-aquae*, *Oocystis borgei*	Direct/Preying ability	[240]
Fungus *Trichaptum abietinum*	*Microcystis aeruginosa*, *M. flos-aquae*, *O. borgei*	Direct/Preying ability	[240]
*Irpex lacteus*, *Trametes hirsute*, *Trametes versicolor*, *Bjerkandera adusta*	*Microcystis aeruginosa*	Direct attack	[241]
**Fish as bio controller**			
Silver carp	*Microcystis aeruginosa*	Grazing	[235]
Bighead carp	*Microcystis aeruginosa*	Grazing	[235]
Tilapia (*Oreochromisni loticus*)	*Microcystis aeruginosa*	Ingestion and digestion	[242]
**Virus as bio controller**			
Virus HaV	*Heterosigma akashiwo*	Direct/Lysis infection	[243]
HaNIV	*Heterosigma akashiwo*	Direct/Induction of cell death or apoptosis	[244]
HcRNAV	*Heterocapsa circularisquama*	Direct/Induction of cell lysis	[245]

#### 4.5.1. Physical Control

Physical methods include control of sources or levels of nutrients entering from industrial or urban sewage, treating wastewater, reduction in ecosystem salinity, preventing water rotation to avoid algal spreading, control of untreated ship ballast water discharge and preventing oil spills at sea, removing deceased fish from coastal water, and filtering the contaminated water by suctioning and returning back the purified water. Mechanical control includes the removal of algal cells by dispersion of clay on the water surface. In Korea and other countries of the world, fish farming is a valued industry, threatened by harmful algal blooms, and the clay method has been widely adopted for the removal of these algal blooms [42].

#### 4.5.2. Biological Control

Living organisms such as microorganisms, aquatic birds, domestic animals, viruses, and bacteria are used as biological control measures to limit populations of phytoplankton [42]. Different microorganisms including bacteria, protozoa, viruses, algae, yeasts, molds, and Rickettsia have been used as biocontrollers for HABs [66,246]. In highly nutrient-loaded lakes, some strains of bacteria control cyanobacterial blooms by producing allelochemicals [247]. The SSZ01 strain of bacteria produced highly toxic β-carbolines (e.g., harmine and norharmane) and showed significant anti-algal activity [248]. Besides bacteria, fungi also secrete some inhibiting agents such as extracellular compounds, which control HABs. White rot *Lopharia spadicea* fungus has been shown to significantly inhibit *Microcystis aeruginosa*, *Glenodinium* spp., and *Cryptomonas ovata* [240].

Myxobacteria are an example of having the potential to kill unicellular filamentous Cladophora by having close contact. Mostly algicidal bacteria secrete algicidal compounds which lyse algae [249]. Palmitoleic acid is produced by a marine *Vibrio bacterium* which is vital for terminating harmful algal blooms in marine ecosystems. It is also reported that BS02 bacteria has an inhibiting effect on *A. tamarense*, showing selective control in algal blooms [247]. However, some fungi are algicidal and can directly kill the algal species, Han et al. pointed out four species of fungi, including *Trametes hirsuta* T24, *Irpex lacteus* T2b, *Bjerkandera adusta* T1. and *Trametes versicolor* F21a, that can degrade algae by attacking them directly. Globally, some important bacterial species are used as biological control of harmful algal blooms; for example, *Microcystis aeruginosa* can be controlled by *Halobacillus* sp. [131], *Streptomyces* sp. [221], *Pedobacter* sp. (MaI11-5) [249], *Pseudomonas putida* [215], *Sandaracinobactor sibiricus* [223], *Agrobacterium vitis* [222], and *Sandaracinobactor neyagawaensis* [219], while Rhodobacteraceae Strain PD-2 can control the *Prorocentrum donghaiense* [218], and *Pseudomonas fluorescens* can control *Heterosigma akashiwo* [214]. Although biological control of HABs is diverse, enormous studies have demonstrated various methods of controlling the HABs using biological applications.

Besides these, some other important strategies opted as biological control include fish species that graze on these blooms and fungi that can decompose by direct attack on them. Viruses have the ability to reproduce quickly and can specifically interact with HAB species and cause their destruction in larger masses. Zooplankton are also voracious grazers of HABs, some of the algae can control HABs by bio-flocculation and various bacterial strains, as discussed in previous sections in detail, can also additionally control HABs by producing secondary metabolites, a cell-to-cell contact mechanism, and by producing antagonistic volatiles, respectively.

#### 4.5.3. Chemical Control

Various chemicals are used to control cyanobacterial harmful algal blooms (cHABs), CuSO_4_ is effective against HABs; however, it is toxic to other plants and animals and its residues are found in sediments and are considered legacy pollutants. Hydrogen peroxide is selectively used against cyanobacteria and possesses no severe side effects on the ecosystem [14,250]. These treatments can be used for limited small areas, whereas, hydrogen peroxide is degraded rapidly by different physio-chemical and biotic activities; thus, its treatment should be applied many times throughout the blooming period. The multiple treatments used for effective results have become costly. Therefore, cautionary measurements for cHABs that produce toxins should be considered because endotoxins released by dead organisms contaminate drinking and irrigation water. Hydrogen peroxide usage is more reliable than copper sulfate because its oxidation stimulant is light and breaks microcystins into peptide residue [250]. It would therefore detoxify water that is affected by microcystin-producing HABs. Phosphorous is immobilized in sediments by chemical precipitation. However, it is not effective because its repeated treatments are expensive. All available forms of nitrogen are soluble in water so no techniques can be used to immobilize it [14]. As the research studies and advanced technologies have revolutionized, however, HAB control is still hampered by several research gaps and effective control measures are immediately required for the mitigation of HABs.

#### 4.5.4. Molecular Approaches

Advanced “-omic” technologies such as proteomics, transcriptomics, metabolomics, and genomics offer a platform to study the HAB species and community dynamics [121]. However, exploring this is still limited because primers are lacking for many important species. However, genomic tools can extend to obtain standard practices and find out the functioning of some genes [218]. Furthermore, identifying the gene regulatory mechanisms for the production of toxins in HAB species is a difficult goal to achieve.

### 4.6. Constraints of HAB Controlling Methods

The effectiveness of controlling methods has some limitations that do not allow these preventive measures to work more precisely. From a brief literature survey, some important limitations are drawn in this section.

#### 4.6.1. Physical Methods

Generally, some of the physical methods (e.g., aeration) are more effective in deep-water columns. However, it highly depends on the rate of airflow and the degree of stratification, and the size of the water body, the larger the water body, the less effective and more costly the methods are. Whereas, hydrologic manipulation needs a sufficient volume of water and a capability for controlling the flow. Oftentimes it can be expensive and unintentional consequences towards other aquatic organisms are expected. Similarly, mechanical mixing has a limited range as operating with individual devices may be away from targeted regions; thus, providing a suitable environment for algal growth in those areas. Desiccation or reservoir drawdown is likely to have a significant impact on other aquatic biota in the system. It could be expensive, as it needs many input resources, especially water to refill the water body. Surface skimming is ineffective until the later stages of bloom. At the later stages of bloom, a lot of harmful aspects have materialized. Therefore, proper equipment is required before the implementation. Ultrasound may be lethal to green algae and zooplankton can disrupt the cellular masses, but the effectiveness depends on the geometry of the species.

#### 4.6.2. Chemical Methods

Algaecides have risks of cell lysis and the direct release of harmful toxins into the targeted environment; therefore, these are usually used at the earlier stages of bloom formation. Some algaecides on the other hand are potentially toxic to other organisms such as fish, invertebrates, and zooplanktons. Barley straw is only effective against new algal species as it can inhibit their growth rather than killing the existing algal species. While, after 2–8 weeks barley straw is seriously harmful to the waterbody as its decay produces harmful chemicals and can cause fish death by deoxygenation. Coagulation processes are limited by depth because the lysis of coagulated cells over time releases the toxins directly into the aquatic ecosystem. Whereas, flocculation is also subject to depth limitations.

#### 4.6.3. Biological Methods

Most of the biological methods including microorganisms and fungi are species-specific, whereas the fish species also cannot kill the toxicant species. In open systems, the application of biological methods may regrettably reinforce the fear of irreversibility. Therefore, this makes it a highly risky strategy. Nevertheless, it could be under consideration only by virtue of its positive outputs. However, the main limitation of introducing the controlling species remains the uncertain conditions. In addition, logistic difficulties in the application of predators, such as the scaling of cultures to obtain a high number of predators and the limitations in their potential use outside the laboratory conditions are also considerable.

## 5. Emerging Technologies and Limitations in Their Application

Harmful algal blooms have sparked a lot of studies around the world. Consequently, numerous emerging advanced technologies are providing breakthroughs to solve the management issues of HABs we are dealing with.

Methods for detecting and quantifying toxins are critical in this regard, and progress has been rapid. For all major HAB toxins, classical analytical techniques combining chromatography and mass spectrometry (e.g., LC-MS) have developed significantly. They are now replacing many older approaches, such as the commonly used but socially unpopular bioassays on mice. Simple kits, on the other hand, that are similar to home pregnancy kits have been produced. These allow for low-cost, quick toxin testing and show a tremendous aptitude for application in the sample screening; thus, eliminating the expensive analysis for large samples in monitoring programs [192]. Bloom detection and tracking are other significant management requirements. At a larger scale, satellite remote sensing is being utilized to detect HABs in the Gulf of Mexico, and forecasts of impending landfall or exposure are now being made using simple transport models [251]. Because the existing blooms identified are mono-specific and dense in nature, hence have a chlorophyll property that shows their existence, that capability is difficult to transmit to other HABs. Remote sensing applications for other HABs, for example, depending on the detection of water masses using the temperature of the sea surface are promising [252]. Satellite-based remote sensing has some limitations: (a) data is limited to the ocean surface, which is typically <15 m but much shallower in coastline waters. This is a serious issue if HABs are intense in distinct subsurface layers; (b) landmasses and cloud cover, limiting its utility in shoreline waters, where the HABs originate; (c) different groupings of phytoplankton are resolved in a limited way from complicated, dynamic communities. For at least the last decade, researchers have been attempting to clarify the pigment-based functional categories or size of phytoplankton with mixed results [253,254,255]. Single or multi-spectral fluorometers, particle size analyzers, absorption, and backscatter sensors are the in-situ methods for quantifying phytoplankton biomass. However, these technologies provide size-based signals in bulk that are not specific to the HAB taxa [256]. Optical, chemical, and molecular technologies are used in other in situ sensing systems on moored and mobile platforms. The Optical Plankton Discriminator (OPD), also known as the Brevebuster, was developed in the late 1990s to detect optical signals of toxic dinoflagellate *K. brevis*, based on its unique pigment signature in mixed phytoplankton communities [257]. The Environmental Sample Processor (ESP), which was designed for moored deployments, detects and quantifies HAB species and toxins using immuno- or molecular probe-based tests [258,259,260]. Over the last decade, a number of field studies based on portable image technologies and various lab methods have been developed. Several of them are now frequently employed in HAB monitoring projects, the best known are FlowCAM and IFCB. FlowCAM detects and monitors the presence of HAB species using the principles of fluidics based on the flow cytometry and the optics of the light microscope. Whereas, to generate the high-resolution images of <10–150 μm particle sizes, IFCB combines flow cytometry and video technology [261]. However, despite progress, accurate and precise identification of phytoplankton species using automated algorithms remains a bottleneck in their use, which frequently needs human assistance [262]. Furthermore, for many HAB species, “molecular probes” have been produced at the lowest scale, allowing algal cells to be identified and counted more readily and quickly than the standard microscopy allows [263]. Biologically unknown toxins, cryptic species, congeners, and pathways might stymie the development of widely used test kits or sensors for poisons and species, as well as the creation of ground-truthing standard sets. Precisely, what defines a “bloom” differs greatly amongst the species of HAB, and many toxin-producers may persist in small numbers. Whereas a toxic “event” is generally derived by regulatory authorities concerned with humans and food safety measures, this metric may not be relevant for researchers and managers working to understand and control HABs before they become intensely dangerous [33]. The availability of more affordable instruments has increased the usage of observation systems, but long-term expenses associated with maintenance, and purchasing proprietary chemicals might be a barrier that is generally unshared among users [33]. In conclusion, the choice of the right technology may necessitate the coordinated employment of complementary techniques, which is useful in management efforts to mitigate and control HABs.

## 6. Conclusions

Harmful algal blooms present a wide range of problems and consequences including their causes and control. Aquatic pollution and anthropogenic interventions in coastal areas have increased their populations drastically and the emergence of novel toxic species. Recently, due to the extensive and steady events of HABs that cause deleterious impacts on aquaculture, human health, tourism, and foremost the entire coastal economy, there is an increasing need for the scientific community to realize the phenomenon, much greater than in the past. Furthermore, changes in global climatic patterns have significantly influenced their persistence and toxicity which is further aggravating the environmental sustainability. In many parts of the world, well-managed fisheries and aquaculture industries, as well as other resources, have been seriously threatened by HABs and this wave of danger will expand for those countries that have not recognized this problem or struggle to encounter it. Although, technological advancements have developed the abilities in ocean monitoring and have opened spaces for the identification of blooms, as well as to explore the chemical, physical, and biological parameters that cause the emergence, expansion, and disappearance of these algal blooms. There are still many loopholes that should be necessarily filled through mutual coordination by research organizations. However, the innovation and creativeness of explorers for future improvements are highly dependent on the collaboration and cooperation of biological and physical researchers who are struggling with these bloom populations.

## Data Availability

Not applicable.
